# Supplementary material for: Intracellular Polyphenol Wine Metabolites Oppose Oxidative Stress and Upregulate Nrf2/ARE Pathway
Source: Antioxidants (Basel). 2022 Oct 19;11(10):2055. doi: 10.3390/antiox11102055 (PMC9598741; doi:10.3390/antiox11102055)
Supplement: Supplementary file 1 [file antioxidants-11-02055-s001.zip › Supplementary Figure S1.pdf]

Supplementary Figure S1.

minimum metabolite level — maximum metabolite level

id	Retention time (min)	m/z(-) detected	m/z(-) expected	elemental formula	Mass Error (ppm)	PUTATIVE IDENTIFICATION	METABOLITE CLASS	p[1]	p[2]	WINE EXTRACT	C5-1	C5-3	C5-5	C10-1	C10-3	C10-5	C20-1	C20-3	C20-5	T5-1	T5-3	T5-5	T10-1	T10-3	T10-5	T20-1	T20-3	T20-5
1	8,880483333	905,25459	905,259	C56H42O12	-4,872074351	resveratrol tetramer	stilbenes and viniferins	0,500248	0,185681	5.184	0	0	0	0	0	0	0	0	0	1.783	5.902	9.979	2.268	7.490	12.748	1.006	3.301	5.712
2	7,430683333	499,13796	499,139	C28H22O6	-2,092000439	epsilon viniferin (formic adduct)	stilbenes and viniferins	0,271712	0,102033	3.091	34	55	84	33	36	31	35	35	34	606	1.864	3.032	697	2.211	3.701	377	1.192	1.875
3	9,276433333	951,25947	951,265	C56H42O12	-5,81447833	resveratrol tetramer (formic adduct)	stilbenes and viniferins	0,196106	0,066008	474	0	0	0	0	0	0	0	0	0	243	983	1.696	286	1.089	1.939	49	330	617
4	8,1806	447,0702						0,195239	0,051556	90	0	4	4	0	0	6	0	0	0	474	1.331	2.064	349	938	1.501	211	579	941
5	8,8176	725,19854						0,17897	0,054582	408	2	10	21	0	0	0	0	2	0	244	894	1.434	249	870	1.597	83	303	473
6	8,492683333	447,07018						0,126759	0,043139	0	0	0	0	0	0	0	0	0	0	147	504	826	113	403	668	77	293	440
7	12,7559	571,28659						0,126001	0,056506	3	0	12	74	0	47	133	0	0	3	30	250	491	150	844	1.452	0	0	0
8	8,0048	499,13798	499,139	C28H22O6	-2,036625798	epsilon viniferin (formic adduct)	stilbenes and viniferins	0,111378	0,045331	465	0	0	0	0	0	0	0	0	0	64	316	505	96	370	611	55	196	333
9	8,555566667	951,25707	951,265	C56H42O12	-8,332480769	resveratrol tetramer (formic adduct)	stilbenes and viniferins	0,102039	0,042238	370	0	0	0	0	0	0	0	0	0	46	210	436	60	311	560	13	111	209
10	7,430683333	359,09074						0,101433	0,039229	422	0	0	0	2	1	0	2	4	6	65	264	433	86	298	502	49	160	261
11	8,432166667	499,13813	499,139	C28H22O6	-1,747786112	epsilon viniferin (formic adduct)	stilbenes and viniferins	0,100677	0,045942	526	0	0	0	0	0	0	0	0	0	54	240	417	82	287	513	47	211	331
12	6,064716667	575,11691	575,118	C30H26O13	-1,901930176	prodelphinidin Pg-P (M-H2O adduct)	flavan-3-ols and	0,099533	0,033693	1.493	0	0	0	0	0	0	0	0	0	45	272	439	53	281	525	0	39	127
13	9,276433333	498,13251						0,097449	0,034024	88	0	0	0	0	0	0	0	0	0	63	232	419	54	274	482	12	95	150
14	6,52355	863,17119	863,182	C45H38O19	-12,51851385	prodelphinidin Pg-P-P (M-H2O adduct)	flavan-3-ols and derivatives	0,097363	0,041932	1.264	0	0	3	2	0	0	0	0	0	23	184	345	62	325	571	5	74	171
15	7,430683333	644,05918						0,094382	0,03489	292	0	0	0	0	0	0	0	0	0	65	222	369	71	241	454	25	122	191
16	8,880483333	1023,1894						0,091681	0,038951	158	0	0	0	0	0	0	0	0	0	22	180	349	59	243	452	12	88	183
17	5,46155	865,19278	865,197	C45H38O18	-4,872385232	procyanidin P3 type	flavan-3-ols and derivatives	0,09135	0,067958	26.740	4	3	2	0	2	4	6	0	0	12	129	205	95	380	663	92	377	636
18	9,02485	587,0805						0,090921	0,024854	0	0	0	0	0	0	0	0	0	0	57	233	421	60	227	394	13	40	94
19	8,5346	725,19709						0,090679	0,037116	188	0	0	0	0	0	0	0	0	0	33	189	360	55	233	416	6	116	174
20	6,62835	861,15941						0,089587	0,015417	655	0	0	1	0	0	5	0	0	8	78	327	542	50	193	355	2	4	24
21	5,388183333	863,17565	863,182	C45H36O18	-7,361673913	procyanidin A- P3 type	flavan-3-ols and derivatives	0,085909	0,029431	2.531	0	0	0	0	0	1	0	0	0	43	184	302	54	224	410	2	19	103
22	8,723266667	906,25733						0,082381	0,031917	90	0	0	0	0	0	0	0	0	0	26	155	291	36	194	367	6	54	112
23	7,74275	435,10691	435,107	C24H20O8	-0,209625504	(Epi)catechin 3-O-coumarate	flavan-3-ols and derivatives	0,081552	0,065701	2.375	0	0	0	0	0	0	0	0	0	47	188	311	64	252	451	137	503	812
24	7,191983333	517,14722	517,134	C25H26O12	25,57278146	vitisin B	flavan-3-ols and derivatives	0,079285	0,03715	1.270	0	0	0	0	0	0	0	0	0	23	146	253	60	197	316	26	127	214
25	8,138683333	725,19963						0,078172	0,032812	1.362	0	0	0	0	0	0	0	0	0	20	151	272	28	165	309	11	65	140
26	8,639416667	545,17849						0,077339	0,038391	373	0	0	0	0	0	0	0	0	0	31	147	239	46	191	302	45	146	248
27	8,337833333	725,19731						0,07685	0,030042	135	0	0	0	0	0	0	0	0	0	30	148	258	28	174	291	9	74	121
28	6,033283333	863,17268	863,182	C45H38O19	-10,80176721	prodelphinidin Pg-P-P (M-H2O adduct)	flavan-3-ols and derivatives	0,075072	0,027652	493	0	2	3	0	0	3	0	0	0	10	138	234	46	179	308	4	40	79
29	4,960816667	577,13407	577,134	C30H26O12	0,122688275	procyanidin P2 type	flavan-3-ols and derivatives	0,073985	0,041906	64.995	0	0	0	0	0	0	0	0	0	24	95	147	65	214	372	45	147	249
30	6,52355	575,11677	575,118	C30H24O12	-2,145197916	Procyanidin P2 A type	flavan-3-ols and derivatives	0,072566	0,019199	338	0	0	0	0	0	0	0	0	0	29	176	264	38	131	262	0	25	44
31	11,01496667	498,28725						0,070849	0,0419	19	12	13	26	14	25	24	15	42	66	49	130	223	60	190	343	35	114	155
32	6,680766667	735,13361						0,06931	0,029235	137	0	0	2	0	0	0	0	0	0	19	120	177	50	174	242	27	105	137
33	7,69035	529,14825						0,064894	0,025673	142	0	0	0	0	0	0	0	0	0	24	103	186	27	121	202	14	63	103
34	6,908983333	575,11734	575,118	C30H24O12	-1,149651985	procyanidin P2 A type	flavan-3-ols and derivatives	0,06318	0,030486	127	0	0	0	0	0	0	0	0	0	7	70	146	25	123	241	12	57	98
35	7,598383333	607,14288						0,061346	0,023597	426	0	0	0	0	0	0	0	0	0	30	135	206	20	94	147	25	70	141
36	8,1806	603,07772						0,061291	0,030461	4	0	0	0	0	0	0	0	0	0	11	66	116	38	131	240	15	64	112
37	7,430683333	265,04873						0,060954	0,024665	148	0	0	0	0	0	0	0	0	0	21	90	156	26	109	184	9	56	89
38	9,077266667	645,17331						0,060317	0,024477	0	0	0	0	0	0	0	0	0	0	15	114	199	19	82	155	11	65	109
39	6,347733333	575,11759	575,118	C30H24O12	-0,718927985	procyanidin P2 A type	flavan-3-ols and derivatives	0,059357	0,028533	385	0	0	0	0	0	0	0	0	0	14	68	116	26	125	209	13	68	99
40	9,378866667	294,06259						0,058666	0,028966	423	40	40	46	33	37	52	34	34	41	39	148	230	63	119	193	57	115	161
41	6,148566667	300,99741	300,998	C14H6O8	-1,96957317	ellagic acid		0,058056	0,004726	5.473	14	7	15	19	5	0	16	6	2	62	144	218	52	85	122	63	73	98
42	9,203066667	499,13888	499,139	C28H22O6	-0,243853538	epsilon viniferin (formic adduct)	stilbenes and viniferins	0,057682	0,03532	29	0	0	0	0	0	0	0	0	0	13	85	146	21	102	180	32	119	203
43	7,732266667	433,11135						0,056469	0,030946	1.931	0	0	0	0	0	0	0	0	0	18	71	130	22	98	169	18	89	146
44	6,127616667	739,16218						0,054643	0,035517	2.379	0	0	0	0	0	0	0	0	0	3	28	109	9	115	194	13	83	136
45	6,3687	477,06654	477,066	C21H18O13	1,138245939	quercetin-O-glucuronide	flavonoids	0,05402	0,009569	10.246	0	6	3	21	0	1	0	0	0	29	129	234	14	63	104	3	27	44
46	6,98235	507,11322	507,113	C22H22O11	0,442677737	rhamnetin-3-rhamnoside (formic adduc	flavonoids	0,053769	0,027679	10.171	0	0	0	0	0	0	0	0	0	23	82	142	20	69	139	23	77	150
47	8,90955	227,07034	227,070	C14H12O3	1,500488281	resveratrol	stilbenes and viniferins	0,052388	0,052248	1.825	49	49	46	47	53	54	49	46	49	65	131	170	87	193	282	163	389	619
48	6,46065	723,16568						0,051719	0,034568	1.805	1	6	0	2	0	2	3	0	0	0	44	74	18	107	194	11	84	138
49	6,69125	723,16475						0,050692	0,031629	932	0	0	0	0	0	0	0	0	0	0	38	90	14	81	169	9	66	108
50	3,1046	164,06973						-0,05133	0,061821	2.070	38	148	240	20	84	145	52	189	325	0	9	16	4	23	40	8	40	76
51	1,843466667	321,04747						-0,05207	0,047472	23	4	135	244	3	54	93	19	173	279	0	0	0	0	0	0	0	1	0
52	1,636216667	243,06072	243,061	C9H12N2O6	-1,162461246	uridine		-0,05493	0,074291	5.445	194	518	766	76	161	240	243	462	671	32	104	167	86	186	285	39	108	161
53	1,73055	180,06463						-0,05559	0,049254	782	52	164	229	34	94	133	81	221	276	0	4	9	5	20	24	10	37	48
54	9,203066667	192,04687						-0,06191	0,082269	0	37	146	235	46	135	229	101	340	557	0	15	28	9	33	58	22	75	132
55	13,37193333	437,28514						-0,06563	0,061955	13	99	314	509	56	159	263	99	286	485	10	89	143	6	12	13	20	48	61
56	2,428066667	346,05402						-0,07218	0,065403	117	87	258	398	72	206	304	127	293	435	0	0	16	2	22	66	2	27	49
57	1,520916667	565,04478						-0,08139	0,076797	54	91	531	974	28	237	385	111	376	706	28	12	20	12	53	166	22	16	17
58	1,832983333	303,08135						-0,0848	0,07167	1	71	297	501	46	170	278	125	420	688	0	0	0	0	6	9	0	0	1
59	8,369266667	1017,5123						-0,08999	0,043425	11	342	794	1.094	163	341	505	190	368	532	89	123	134	75	41	26	69	116	125
60	2,5748	267,07166	267,072	C10H12N4O5	-1,265812343	arabinosylhypoxanthine		-0,09048	0,309212	186	999	2.966	4.620	957	2.512	3.409	1.250	3.225	4.417	381	1.210	1.811	811	2.293	3.094	561	1.807	2.543
61	9,203066667	333,08973						-0,09127	0,123033	0	84	315	517	96	302	529	238	737	1.206	0	25	64	16	82	139	48	179	289
62	1,541883333	346,05407						-0,11723	0,108734	376	341	766	1.213	236	564	915	318	781	1.220	25	34	116	36	121	217	36	90	174
63	5,409133333	1652,8262						-0,18756	0,178765	0	185	1.710	3.576	11	431	1.517	169	1.728	3.944	0	9	0	0	4	3	2	2	4

Figure S1. List of the metabolites that characterize the treated and control cells, based on p[1] loading values of Principal Component Analysis of Figure 1. Legend: Based on the loadings (P[1]) of the Principal Component Analysis of Figure 1, arbitrary thresholds p[1] >0.05. and p[1]< -0.05 were chosen as characterizing respectively treated cells (T) and control cells (C). The relative quantification in all the samples and in the wine extract, analysed as reference, are reported in arbitrary units. The relative amount of each metabolites in all the cell samples are shown as heat map with blue=sample with lowest metabolite level; yellow= sample with average metabolite level; red=sample with highest metabolite level. The abundance of the metabolites in the wine sample are indicated with the same heat map. UI=unidentified. 5, 10, 20: millions of cells collected; 1,3,5: µl of cell extract analysed by UPLC-ESI-MS.
