# Supplementary material for: Intracellular Polyphenol Wine Metabolites Oppose Oxidative Stress and Upregulate Nrf2/ARE Pathway
Source: Antioxidants (Basel). 2022 Oct 19;11(10):2055. doi: 10.3390/antiox11102055 (PMC9598741; doi:10.3390/antiox11102055)
Supplement: Supplementary file 1 [file antioxidants-11-02055-s001.zip › Supplementary Figure S2.pdf]

Supplementary Figure S2.

id	Retention time (min)	m/z(-) detected	m/z(-) expected	elemental formula	Mass Error (ppm)	PUTATIVE IDENTIFICATION	METABOLITE CLASS
1	6,306233333	577,1326728	577,134	C30H26O12	-2,30	procyanidin P2 type	flavan-3-ols and derivatives
2	8,327583333	435,1063198	435,107	C24H20O8	-1,56	epicatechin 3-O-coumarate	flavan-3-ols and derivatives
3	6,264316667	435,1279637	435,129	C21H24O10	-2,38	(epi)catechin-O-desoxyhexoside	flavan-3-ols and derivatives
4	7,1609	517,1474844	517,134	C25H26O12	26,08	vitisin B	flavan-3-ols and derivatives
5	4,940383333	577,1322405	577,134	C30H26O12	-3,05	procyanidin P2 type	flavan-3-ols and derivatives
6	5,4411	865,1892923	865,197	C45H38O18	-8,91	procyanidin P3 type	flavan-3-ols and derivatives
7	7,701133333	435,1068819	435,107	C24H20O8	-0,27	catechin 3-O-coumarate	flavan-3-ols and derivatives
8	4,607383333	289,0698883	289,071	C15H14O6	-3,85	catechin	flavan-3-ols and derivatives
9	5,25245	289,0703573	289,071	C15H14O6	-2,22	epicatechin	flavan-3-ols and derivatives
10	5,25245	287,0537523	287,055	C15H12O6	-4,35	dehydrokaempferol	flavonoids
11	6,52395	303,0513776	303,05	C15H12O7	4,55	taxifolin	flavonoids
12	7,69065	433,1121388	433,113	C21H22O10	-1,99	trihydroxy flavone O-hexoside	flavonoids
13	9,588616667	285,0383897	285,039	C15H10O6	-2,14	kaempferol	flavonoids
14	9,7644	315,0488254	315,05	C16H12O7	-3,73	rhamnetin	flavonoids
15	8,513866667	301,033906	301,034	C15H10O7	-0,31	quercetin	flavonoids
16	4,96135	179,0324983	179,034	C9H8O4	-8,39	caffeic acid	hydroxycinnamic acids
17	5,899866667	163,0376463	163,039	C9H8O3	-8,30	coumaric acid	hydroxycinnamic acids
18	4,471133333	299,0753041	299,076	C13H16O8	-2,33	hydroxybenzoic acid hexoside	hydroxybenzoic acids
19	7,96315	499,1374956	499,139	C28H22O6	-3,01	e-viniferin (formic adduct)	stilbenes and viniferins
20	8,390466667	499,1376972	499,139	C28H22O6	-2,61	e-viniferin (formic adduct)	stilbenes and viniferins
21	7,80595	227,069441	227,07	C14H12O3	-2,46	resveratrol isomer	stilbenes and viniferins
22	9,161283333	453,1326046	453,133	C28H22O6	-0,87	e-viniferin	stilbenes and viniferins
23	9,337083333	499,1382343	499,139	C28H22O6	-1,53	e-viniferin (formic adduct)	stilbenes and viniferins
24	7,399566667	499,1373876	499,139	C28H22O6	-3,23	e-viniferin (formic adduct)	stilbenes and viniferins
25	8,859733333	227,0698517	227,07	C14H12O3	-0,65	resveratrol	stilbenes and viniferins
26	8,828283333	905,2539639	905,259	C56H42O12	-5,56	resveratrol tetramer	stilbenes and viniferins

Figure S2. Mass spectral features of wine extract metabolites accumulated in treated THP-1 cells and shown in Figure 2. Legend: the retention time, detected m/z value (negative ionization mode), expected m/z value, elemental formula, mass error in ppm, putative identification and metabolite class are reported. Putative identification were supported by isotopic similarity and fragmentation.
